# Supplementary material for: The effects of carbon emissions, rainfall, temperature, inflation, population, and unemployment on economic growth in Saudi Arabia: An ARDL investigation
Source: PLoS One. 2021 Apr 5;16(4):e0248743. doi: 10.1371/journal.pone.0248743 (PMC8021147; doi:10.1371/journal.pone.0248743)
Supplement: Attachment — Submitted filename: Responses of Reviewers.docx [file pone.0248743.s001.docx]

**RESPONSES TO REVIEWERS**

**PONE-D-20-37757**

**Manuscript title:** The effects of carbon emissions, rainfall, temperature, inflation, population, and unemployment on economic growth in Saudi Arabia: an ARDL investigation

**Reviewer #1:** This is an interesting manuscript that covers a range of climate change, social and macroeconomic issues with reference to Saudi Arabia. It discusses an important cluster of numerous questions in Saudi Arabia. I include several comments that should be addressed before publishing this manuscript. Some comments are specific, other are general. The details are given bellow:

Response: We express thanks to Reviewer #1 for kindly reviewing the paper and getting back to us with some positive and helpful comments. We have addressed all the comments of Reviewer #1, so please see below how and where in the revised manuscript we have addressed the comments:

1. Length. This paper is very long. In particular, Introduction and review of literature section should be compact and arranged the citation ascending or descending order.

Response: The introduction and literature sections have now been made compact. The citations have also been rearranged throughout the manuscript according to ascending order of the year. Since PLOS ONE publishes papers in the form of online we thought a relatively long paper would not be a problem. In its current and revised form, it has just over 9000 words, however, we could reduce it by around 1000 words, particularly in the literature review. Keeping in mind that an extensive literature review is helpful for a larger audience we will await further comment on this.

2. The English is better but still requires major editing.

Response: The revised version has been thoroughly edited by a professional English editor who mainly addressed clarity, syntax and grammar issues. When you look at the revised paper where track changes are kept on, you will notice that a professional English editor, namely Phillip Thomas has kindly completed this task for a fee.

3. Data. Annual Time series data is used from the 1990 to 2019. Data is taken from multiple sources; hence, the base year of data set is not similar. You should mention how you equated the base year.

Response: Data used in the study are extracted from only two sources: The World Bank and General Authority of Meteorology and Environmental Protection, Saudi Arabia. We extracted data on all seven macroeconomic and environmental variables, including GDP growth (GDPG), Inflation (INF), Unemployment (UNEMP), Population (POPL), Rainfall (RF), Temperature (TEPT), and Carbon dioxide (CO_2_) directly from their online open access sources without any changes to them. Hence the base year considered for all the whole data set is 1990 and this is consistent for all seven variables. Data on all the variables prior to 1990 and after 2019 were not found available, which is beyond our control. We hope the reviewer will kindly understand this unavoidable circumstance.

4. Overall results section is good, but there is need to add some recent studies for cross citation.

Response: In results and discussion section, we have now added some recent studies for cross citation, which are updated to 2020.

5. Add some policy measures that should be based on results

Response: Based on the key results we have now added some policy actions in the revised version of the paper. We included them after Conclusions section, so please see our effort in section 6, titled Policy Actions, page 19.

**Reviewer #2:** In this manuscript authors tried to explore the possible relationships among several climatic-social dimension with economic development.

1. Overall, the manuscript is poorly written and structured. I am afraid, in its current form, it may not be able to create an interest in readers for global audience.

Response: While Reviewer #2 is not convinced by our work, we are highly appreciative of the review work s/he undertook for us. We sincerely believe Reviewer #2 will now be convinced by the additional review that we have undertaken in the revised version. Particularly, we have given the paper a thorough English editing by a professional editor, and it has been restructured following the review comments from both you and Reviewer #1. With this we believe the paper would create an interest in readers for global audience.

2. Abstract, author did not set up a reasonable background of the research objective – the reason why this research is important.

Response: We have now provided a reasonable background of the research objective in the abstract as well as in introduction section, where we have also addressed the issues of clarity, rationale, problem statement, and objectives of the research.

3. In the introduction, the authors failed to establish a testable hypothesis and interdependencies of the selected variable with the GDP or economic growth.

Response: In the Introduction section, we have now outlined the research questions that this study addresses, and the research outcomes that are expected from this study. Introduction section has now been substantially revised to address this comment. Moreover, interdependencies of the selected variables with the GDP growth have now been discussed in greater detail in literature review section.

4. Methods and Results are poor and do not fit well. There is no discussion and inferences derived from the study.

Response: We have revised the Methods and Results section once again to establish the link between them. In the current version of the paper, discussion followed each key finding so there is not any separate section devoted to discussion. However, we have now fine-tuned the discussion of each of the key findings. In conclusion section, we derived some inferences based on the key findings. As suggested by Reviewer #1, we have also included in page 19 a new section, namely Policy Actions based on the key findings.
